# The effects of aberrant expression of LncRNA DGCR5/miR‐873‐5p/TUSC3 in lung cancer cell progression

**DOI:** 10.1002/cam4.1566

**Published:** 2018-05-23

**Authors:** Judong Luo, Hong Zhu, Hua Jiang, Yayun Cui, Mengjie Wang, Xinye Ni, Changsheng Ma

**Affiliations:** ^1^ Department of Oncology The Affiliated Changzhou No. 2 People’s Hospital of Nanjing Medical University Changzhou China; ^2^ Department of Radiation Oncology Shandong Cancer Hospital and Institute Shandong Cancer Hospital Affiliated to Shandong University Shandong Academy of Medical Sciences Jinan China; ^3^ Department of Radiation Oncology Minhang Branch of Cancer Hospital of Fudan University Shanghai China; ^4^ Department of Radiation Oncology The Affiliated Provincial Hospital of Anhui Medical University Hefei China; ^5^ Department of Radiotherapy The Affiliated Changzhou No. 2 People’s Hospital of Nanjing Medical University Changzhou China

**Keywords:** cancer biology, cancer genetics, lung cancer, radiation therapy

## Abstract

Lung cancer is the most common cause of cancer‐related mortality worldwide, and nonsmall cell lung cancer (NSCLC) accounts for 80% of all pulmonary carcinomas. Recently, long noncoding RNAs (lncRNAs) have been paid attention for exploring treatment of various diseases. Upregulation of DiGeorge syndrome critical region gene 5 (DGCR5) predicts better lung squamous cell carcinoma prognosis; therefore, we explore the role of DGCR5 in lung cancer in our present study. Consecutive patients with LC were treated in our hospital between January 2015 and January 2016. qRT‐PCR demonstrated that DGCR5 was significantly lower in neoplastic tissues than in non‐neoplastic tissues. For in vitro experiments, cell growth, migration, and invasion were significantly lower in A549 cells transfected with pcDNA3.1‐DGCR5 than pcDNA3.1, which were verified by 5‐diphenyltetrazolium bromide (MTT) assay, scratch test, and transwell assay, respectively, with no significant induction on cell apoptosis that was demonstrated by flow cytometry (FCM) assay. Bioinformatics analysis predicted that 3’ untranslated region (UTR) of tumor suppressor candidate 3 (TUSC3, 49‐55 bp) and DGCR5 (801‐807 bp) shared a common hsa‐miR‐873‐5p binding site, and the direct interaction between DGCR5 and hsa‐miR‐873‐5p or hsa‐miR‐873‐5p and TUSC3 was verified by dual‐luciferase reporter assay. qRT‐PCR demonstrated that hsa‐miR‐873‐5p was dramatically higher and TUSC3 was significantly lower in neoplastic tissues than in non‐neoplastic tissues. DGCR5 decreased the protein level of TUSC3 by miR‐873‐5p which was demonstrated by Western blot and immunofluorescence. The role of DGCR5 in tumorigenesis in vivo was consistent with in vitro assays, Ki‐67‐positive cell number (exhibited by immunohistochemical staining), tumor size, and tumor weight of A549‐DGCR5 group were significantly lower in comparison with A549‐control group.

## 1 INTRODUCTION

Lung cancer (LC) is the most common cause of cancer‐related mortality worldwide and includes nonsmall cell lung cancer (NSCLC) and small cell lung cancer (SCLC), of which NSCLC accounts for 80% of all pulmonary carcinomas.^1^, ^2^ Despite the advances in therapies for NSCLC, the mortality rate of patients with NSCLC has not significantly lowered recently^3^; the 5‐year overall survival rate is still <10%, surprisingly, diagnosis followed by surgery at early stage of disease has the ability to generate the 55%‐80% 5‐year survival rate.^4^ The currently existing therapies, including radiotherapy, chemotherapy, and the emerging target therapy, remain unsatisfactory for improving the therapeutic efficacy of patients with lung cancer.^5^ Consequently, it is worth understanding the mechanisms of tumor progression, and it is of great importance in early detection, prevention, and exploring effective targeted treatment strategies of LC.

Noncoding RNAs (ncRNAs) include transfer RNA (tRNA), ribosomal RNA (rRNA), small nuclear RNA (sn RNA), and small nucleolar RNA (sno RNA).^6^ Recently, the attention from short ncRNAs has shifted to long noncoding (lncRNAs), and it has been evident that mammalian genomes principally encode lncRNAs whose function is becoming more and more prominent.^7^ LncRNAs (≥200 bp), which lack significant protein‐coding open reading frames, are pervasively transcribed from intergenic/intronic regions of human genome and have the ability to regulate gene expression at a variety of levels, including chromatin modification, transcription, and post‐transcription.^8^, ^9^ As of January 2016, 294 LncRNAs have been functionally annotated in LncRNAdb (a database of literature described LncRNAs), and majority of them (183 LncRNAs) have been described in humans.^10^, ^11^

It was suggested that lncRNAs could function sponges which regulate levels and activities of microRNAs (miRNAs).^12^, ^13^ Meanwhile, miRNAs (approximately 22 bp) were reported to be primary regulators of gene expression via targeting 3’‐UTR of target genes^14^ and controlling the translation of mRNA into proteins.^15^ Moreover, miRNAs participated in regulating various biological processes in numerous cancers.^16^

Downregulation of DGCR5 in tissues and serum was correlated with poor prognosis of hepatocellular carcinoma^17^; reciprocal regulation of DGCR5 and miR‐320a influenced pancreatic ductal adenocarcinoma cellular malignant phenotype.^18^ Therefore, this study aimed to investigate the function of DGCR5 in LC and identify the miRNAs that could be targeted by DGCR5 as well as the target genes that could be targeted by miRNAs, eventually providing a novel therapeutic target for LC.

## 2 MATERIALS AND METHODS

### 2.1 Participants

Consecutive patients with NSCLC treated in our hospital between January 2015 and January 2016 were studied in our present research. We obtained lung tissue samples that were both neoplastic and non‐neoplastic from patients during the period of operation. The extracted tissue samples were stored immediately at −80°C. All of the patients in the present study provided informed written consent for their participation before surgery and in our research. Ethical permission was also provided for the study.

### 2.2 Cell culture

Human lung epithelial cells (BEAS‐2B), human NSCLC cell line A549, and human kidney cells (293T) were purchased from American Type Culture Collection (ATCC). BEAS‐2B and A549 were cultured in the RPMI‐1640 Medium (Invitrogen, Carlsbad, CA, USA), 293T cells were cultured in Dulbecco’s modified Eagle’s medium (DMEM) (Invitrogen). All medium was supplemented with 10% fetal bovine serum (FBS, Fisher, New York, NY, USA), 100 units/mL penicillin, and 100 μg/mL streptomycin at 37°C under 5% CO_2_ and 95% humidity.

### 2.3 qRT‐PCR

The miRNeasy Mini Kit (Qiagen, Valencia, CA, USA) was used to extract total RNA from tissues and cells in accordance with the manufacturer’s instructions. Concentration and quality of RNA were measured with NanoDrop 2000 (Thermo Fisher, Wilmington, DE, USA). The first‐strand cDNA was synthesized by TransScript first‐strand cDNA synthesis SuperMix (TransGen, Beijing, China) in accordance with the manufacturer’s instructions. RT‐PCR assay was performed by SYBR green qPCR SuperMix (Applied Biosystems Life Technologies, Foster, CA, USA) in ABI prism 7500 sequence detection system (Applied Biosystems Life Technologies). Conditions were presented as below: 55°C for 10 minutes, 40 cycles of 95°C for 30 seconds, 55‐59°C 30 seconds, and 72°C for 42 seconds. Fold changes of each gene were calculated by 2^−ΔΔCt^ (cycle threshold), and expression levels of miRNA and lncRNA/target gene were normalized by U6 and GADPH, respectively.

### 2.4 Cell proliferation assay

5‐diphenyltetrazolium bromide (MTT) assay was adopted to evaluate the cell proliferation changes in different groups. Briefly, cells were seeded into 96‐well plates at the density of 5 × 10^3^/well. Next, cells were incubated with 100 μL 0.5 mg/mL MTT for 4 hours at 37°C, and precipitate was dissolved in 150 μL dimethyl sulfoxide (DMSO). The optical density at 570 nm was evaluated after shaking for 10 minutes.

### 2.5 Flow cytometry assay

At 72 and 96 hours after cell transfection, A549 cells were collected, resuspended in 500 μL precold 1× binding buffer, mixed with 5 μL Annexin V‐fluorescein isothiocyanate (FITC) and 2.5 μL propidium iodide (PI), and finally determined with a FACSAria Sorter (Becton Dickinson, San Jose, CA, USA). The scatter diagram was distributed as followed: Q4: healthy cells (FITC‐/PI‐); Q3: apoptotic cells at an early stage (FITC+/PI−); Q2: apoptotic cells at an advanced stage (FITC+/PI+). Apoptosis rate was calculated as ratio of apoptotic cells in Q3+ Q2 to total cells.

### 2.6 Scratch wound healing assay

A549 cells (8 × 10^5^) were allowed to grow to 100% confluence in 6‐well plates and incubated with 8 μg/mL mitomycin C for 3 hours to inactivate cell proliferation. Confluent cells were subsequently scratched by a 10 μL tip, thereafter, incubated in an incubator at the temperature of 37°C with 5% CO_2_ and 95% humidified atmosphere for 24 hours. The pictures of migration area from fields of control group and treatment group were captured with an inverted microscope and analyzed by Image J.

### 2.7 Transwell assay

Cell invasion assay of A549 cells was carried out in 24‐well plates by transwell chambers (Corning Inc., Corning, USA) which were fitted by a polyethylene terephthalate filter membrane with 8 μm pores. Cells (5 × 10^4^) were placed into serum‐free medium in the upper Matrigel‐coated chamber, while the lower chamber was filled with medium that contains 10% FBS. After incubation of cells at 37°C for 24 hours, cells in the upper chamber were removed away by a cotton swab, while cells traversed to reverse face of the membrane were fixed by methanol and stained with crystal violet. At last, images were captured from 5 randomly chosen fields by a microscope.

### 2.8 Transient overexpression of DGCR5 and miRNA transfection in A549 cells

Full‐length DGCR5 cDNA was amplified from cDNA of BEAS‐2B and cloned into pcDNA‐3 plasmid. The transient overexpression of DGCR5 was achieved by transfection of pcDNA‐3‐DGCR5 into A549 using Lipofectamine 2000 (Invitrogen), and A549 cells transfected with empty plasmid served as control group.

For miRNA transfection, miR‐873‐5p mimics or miR‐negative control (NC) mimics or miR‐873‐5p inhibitor or miR‐NC inhibitor (Thermo Fisher, Waltham, MA, USA) was transfected into indicated A549 cell line using lipofectamine 3000 (Invitrogen) for 72 hours and then analyzed by qRT‐PCR.

### 2.9 Luciferase activity assay

Oligonucleotides containing DGCR5 cDNA fragment including microRNA binding sites was amplified and cloned into the pmirGLO plasmids (Promega, Madison, WI, USA). Mutant DGCR5 (pmirGLO‐DGCR5‐MUT) was generated by site‐directed mutagenesis PCR with platinum pfx DNA polymerase according to the product manual and served as negative control. Luciferase reporter plasmids and target miR‐873‐5p mimics or miR‐NC mimics were cotransfected into cells by Lipofectamine 2000. At 48 hours after transfection, relative luciferase activity was examined in a luminometer by Dual‐Luciferase Reporter Assay System (Promega).

### 2.10 Western blotting

Samples (15 μg protein/lane) were electrophoresed on SDS‐PAGE, next, transferred onto polyvinylidene difluoride membranes by iBlot Gel Transfer Device (Thermo Fisher). The membranes were blocked by Blocking One (Nacalai Tesque, Kyoto, Japan), thereafter, incubated with primary antibodies at 4°C overnight. Afterward, these protein bands were incubated by horseradish peroxidase (HRP)‐conjugated secondary antibodies (Cell Signaling Technology). Bands were treated with ECL Prime Western Blotting Detection Reagents (GE Healthcare Life Sciences, Little Chalfont, UK). Finally, ImageQuant TL GE Healthcare Life Sciences) was used to digitize the band strength.

### 2.11 Immunofluorescence staining

Cells were seeded onto glass coverslips (0.17 mm thickness, 14 mm diameter) in 6‐well plates overnight, thereafter, treated with TMZ for 3 days. Afterward, cells were washed by PBS for 3 times, fixed by 4% paraformaldehyde for 30 minutes, permeated with 0.1% Triton X‐100 for 5 minutes, and blocked by 2% bovine serum albumin (BSA) for 30 minutes. Then cells were incubated with primary antibody which was diluted in 2% BSA at 4°C overnight, after 3 times of PBS rinse, fluorescent secondary antibody was added and incubated at room temperature for 1 hour in the dark place. Coverslips were mounted on slides by mounting medium (Santa Cruz, USA) containing DAPI DNA counterstain. Images were captured by microscopy (IX‐70, Olympus, Japan).

### 2.12 Construction of A549‐DGCR5 cells with stable overexpression of DGCR5

Full‐length DGCR5 cDNA was amplified from cDNA of BEAS‐2B and cloned into pLVX‐Puro plasmid. The lentivirus particles were packaged in 293T cell with Lenti‐X^™^ HT Packaging System (Clontech). The lentivirus was harvested and then infected A549 cells. The A549 cells with successful transfection of pLVX‐Puro‐DGCR5 or empty vector were screened using puromycin. The stably overexpressed DGCR5 in A549 cells were adopted in nude mice.

### 2.13 Determination of anticancer effect of DGCR5 in nude mice

Female BALB/c nude mice (8‐week) were fed at Second People’s Hospital of Changzhou, Nanjing Medical University. Mice were kept under specific pathogen‐free (SPF) conditions with 12‐hours light/dark cycle and free access to autoclaved food and water. Experiment protocol was approved by Second People’s Hospital of Changzhou, Nanjing Medical University Animals Research Committee. Mice were randomly divided into control group and DGCR5 group (n = 5). A549‐control or A549‐DGCR5 cells (3 × 10^6^) were suspended in 0.25 mL PBS and Matrigel (BD Biosciences) mixture at the volume proportion of 1:1, which was inoculated into the right flank of nude mice of control group or DGCR5 group, respectively.

Tumor volume (mm^3^) was measured by a caliper and then assessed by the following formula: 1/2 × length × width^2^. After 8 weeks of tumor growth, the mice were sacrificed and the tumors were removed from mice with a scalpel. The tumor weight was obtained by a electronic balance.

### 2.14 Immunohistochemical staining

LC tissue sections from nude mice were firstly dried for 1 hour at 60°C, secondly dewaxed in xylene, and rehydrated by graded concentrations of alcohol. Antigen retrieval was treated by citrate buffer (pH 6.0) and autoclaved for 90 seconds at 121°C. After washing by PBS, sections were blocked in goat serum (Boster, Wuhan, China) for 30 minutes at room temperature. Subsequently, sections were incubated with Ki67 antibody (Bioss Antibodies, Inc, 1:200) overnight at 4°C or Colorimetric TUNEL Apoptosis Assay Kit (Beyotime, Shanghai, China) at 37°C for 60 minutes. Next, after washing by PBS, sections were incubated with Polink‐1 HRP DAB Detection System One‐step polymer detection system (ZSGB‐BIO, Beijing, China) for 20 minutes at room temperature. Finally, slides were counterstained with hematoxylin.

### 2.15 Statistical analysis

Data were expressed as mean ± SD. Comparisons between 2 groups and multiple groups were performed by Student’s *t* test and one‐way analysis of variance, respectively. *P* < .05 was considered as statistical significance. Analyses in our study were carried out by SPSS version 13 (Chicago, IL, USA).

## 3 RESULTS

### 3.1 DGCR5 was downregulated in LC patient’s tissues

**Figure 1:**
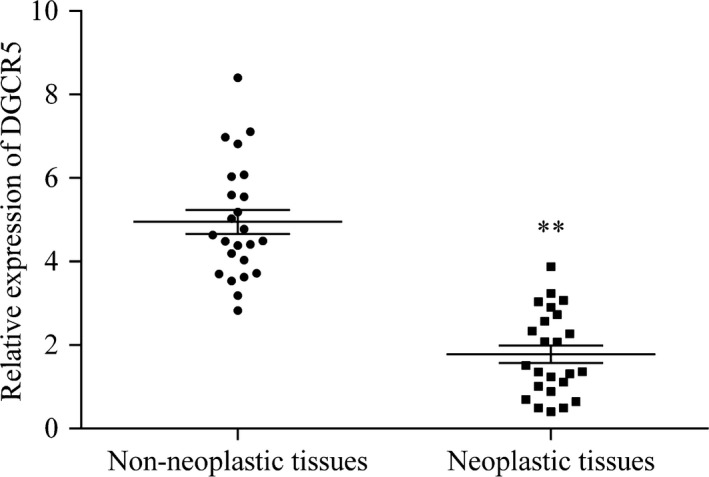
Aberrant expression of DGCR5 in LC tissues compared with normal tissues. The expression of DGCR5 in lung tissue samples of neoplastic and non‐neoplastic was detected by RT‐qPCR. **P < .01 compared with non‐neoplastic lung tissues

To evaluate potential prognostic effect of DGCR5 in LC, its expression status was determined by qRT‐PCR in lung tissue samples that were both neoplastic and non‐neoplastic from 24 patients. Results demonstrated that DGCR5 was expressed at a relatively lower level in neoplastic tissues than in the non‐neoplastic tissues (*P* < .01, Figure 1).

Meanwhile, the correlation between DGCR5 level and multiple clinicopathologic features (age, gender, lymph node metastasis, distant metastasis, and tumor size) was analyzed. We found that high expression of DGCR5 was significantly associated with small tumor size, low incidence of both lymph metastasis and distant metastasis (*P* < .05) with no significant correlation between DGCR5 and the remaining indexes (Table 1).

**Table 1:** The correlation between DGCR5 expression (ΔCt normalized to GAPDH) and clinicopathological factors of patients with LC

Characteristics	No. of patients (%)	DGCR5	
		Mean ± SD	*P* value
Total no. of patients	24		
Age (y)			
>60	15 (62.5)	10.43 ± 0.63	.346
≤60	9 (37.5)	10.18 ± 0.59	
Sex			
Male	14 (58.3)	10.29 ± 0.92	.753
Female	10 (41.7)	10.19 ± 0.42	
Lymphatic metastasis			
N0	16 (66.7)	10.22 ± 0.74	.005
N1‐N3	8 (33.3)	9.34 ± 0.38	
Distal metastasis			
M0	21 (87.5)	10.20 ± 0.53	.007
M1	3 (12.5)	9.57 ± 0.39	
Size (cm)			
>3	13 (54.2)	9.73 ± 0.46	.029
≤3	11 (45.8)	10.24 ± 0.61	

Taken together, these results suggested that DGCR5 might be a tumor suppressor in LC.

### 3.2 DGCR5 inhibited proliferation of LC cells

**Figure 2:**
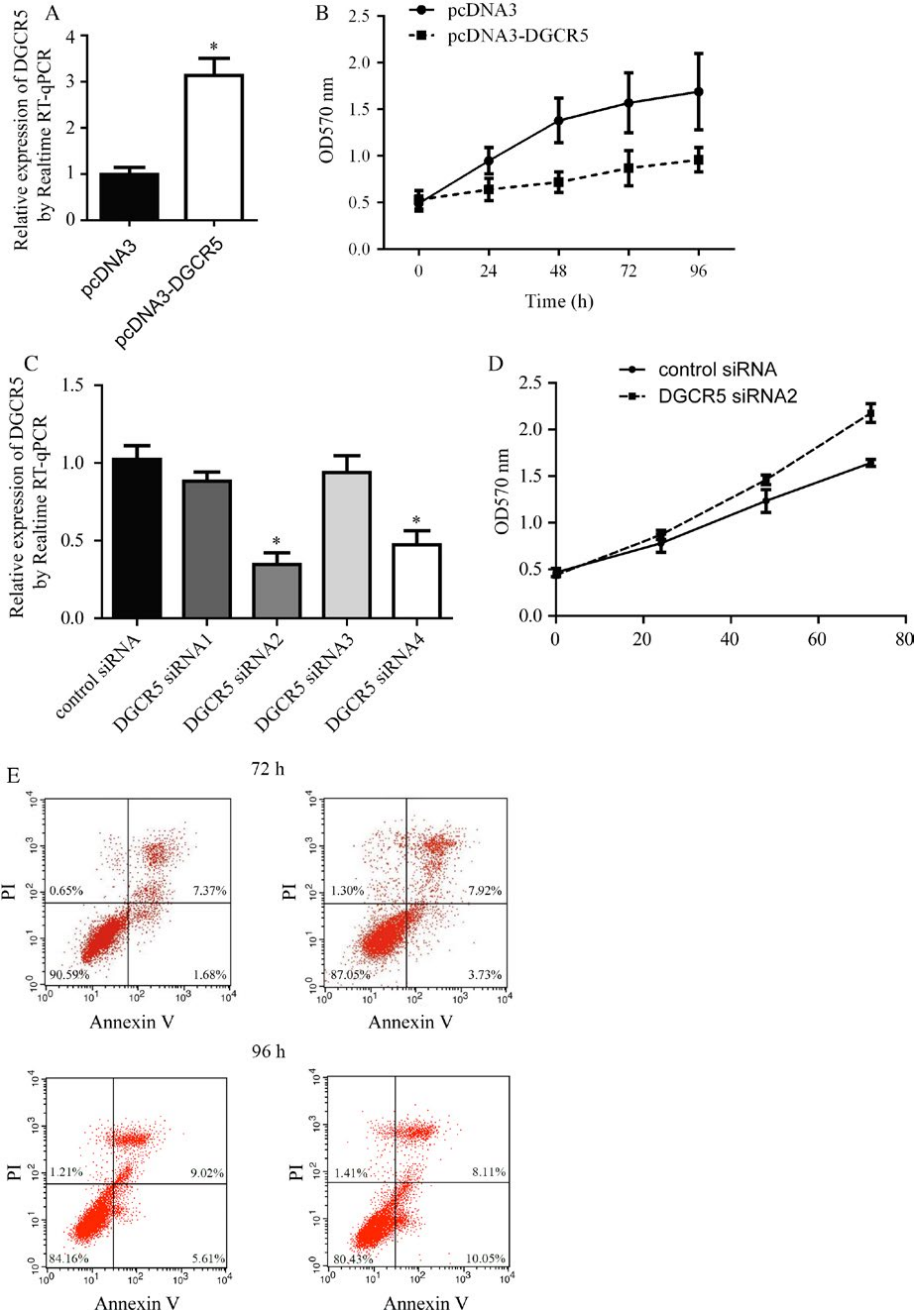
DGCR5 suppressed lung cancer cell proliferation without interference of cell apoptosis. A, Over‐expression of DGCR5 by transfection of pcDNA3‐DGCR5 in A549 was detected by RT‐qPCR. **P < .01 compared with pcDNA3 group. B, Proliferation of A549 cells was greatly suppressed by DGCR5 over‐expression. **P < .01 compared with pcDNA3 group. C, There was a decrease of DGCR5 expression in A549 by treatment of DGCR5 siRNAs, especially DGCR5 siRNA2. *P < .05 compared with control siRNA group. D, Silencing of DGCR5 promoted cell proliferation of A549. **P < .01 compared with control siRNA group. E, Representative images of cell apoptosis assay. Cell apoptosis was not affected by DGCR5 over‐expression in A549

A549 cells were transfected with pcDNA3.1 (control group) and pcDNA3.1‐DGCR5 (experimental group). DGCR5 expression status was detected by qRT‐PCR, results demonstrated that DGCR5 was dramatically higher in A549 cells transfected with pcDNA3.1‐DGCR5 than in A549 cells transfected with pcDNA3.1 indicating our successful overexpression of DGCR5 in A549 cells (Figure 2A, *P* < .01). Moreover, forced overexpression of DGCR5 greatly reduced cell proliferation of A549 (Figure 2B).

For further validation of DGCR5’s role in lung cancer, A549 cells were transfected with DGCR5 control siRNA (control group) and DGCR5 siRNA1‐4 (experimental groups). After the examination of DGCR5 level by qRT‐PCR, we found that DGCR5 was dramatically lower in A549 cells transfected with DGCR5 siRNA2 (*P* < .01) and DGCR5 siRNA4 (*P* < .05) than in A549 cells transfected with DGCR5 control siRNA (Figure 2C). Consequently, DGCR5 siRNA2 which showed the best effects on interfering DGCR5 expression was selected for the following experiments. Conversed to DGCR5 overexpression, silencing of DGCR5 significantly promoted A549 cell proliferation (Figure 2D). The altered cell growth might be a consequence of cell death, so we next sought to detect cell apoptosis after DGCR5 overexpression. However, we did not observe significant apoptosis in response to DGCR5 overexpression in A549 cells (Figure 2E).

In conclusion, these data suggested the involvement of DGCR5 in the cell proliferation of lung cancer cells.

### 3.3 DGCR5 inhibited migration and invasion of LC cells

**Figure 3:**
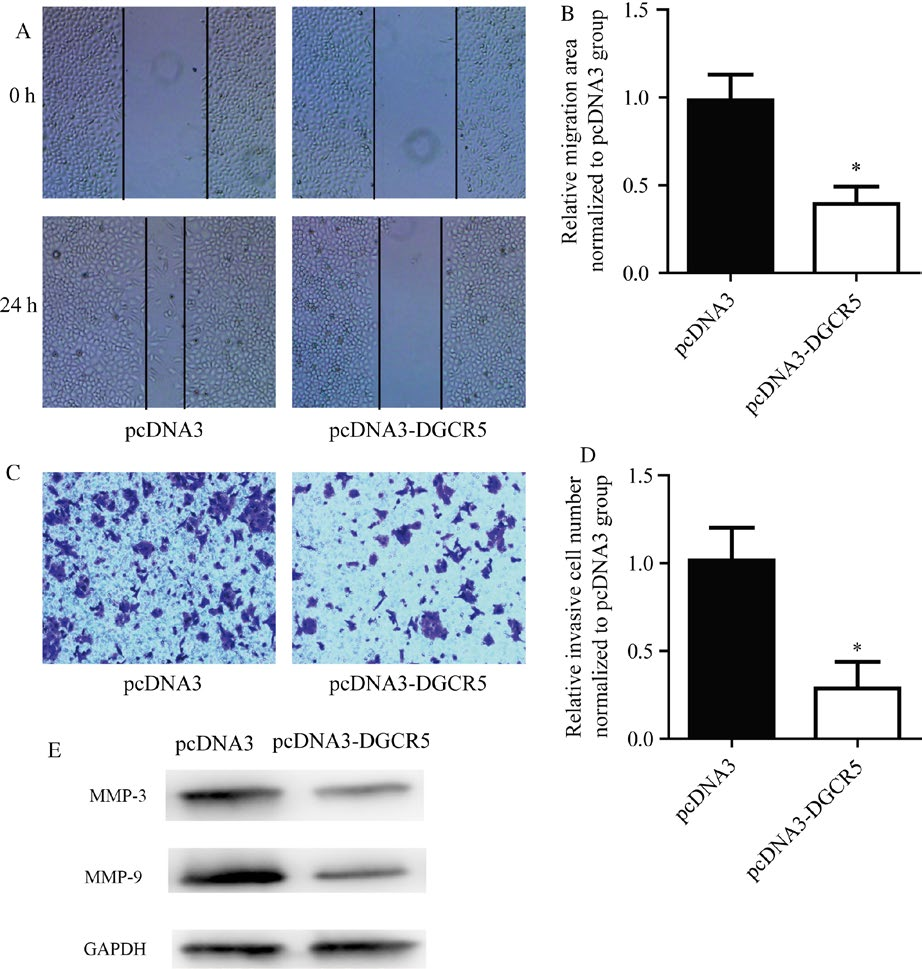
DGCR5 inhibited cell motility of lung cancer cells. A and B, Representative images and quantitative analysis of cell migration assay. DGCR5 over‐expression induced a delayed closure of A549 cells. C and D, Representative images and quantitative analysis of cell invasion assay. E, DGCR5 over‐expression greatly suppressed A549 cells from invaded through membrane

As we observed a negative correlation between DGCR expression with metastasis in patients with lung cancer, we next focus on the role of DGCR5 on lung cell migration and invasion. The migration and invasion ability of A549 cells transfected with pcDNA3‐DGCR5 or empty plasmid were evaluated by scratch wound healing assay and transwell assay, respectively. Results indicated that pcDNA3‐DGCR5 markedly inhibited migration and invasion ability of A549 cells when compared with pcDNA3.1 (Figure 3A‐D, *P* < .01). Moreover, pcDNA3‐DGCR5 also leads to significant expression in migration and invasion‐related marker MMP‐3 and MMP‐9 (Figure 3E).

These data suggested that DGCR5 played an important role in regulating metastasis ability of lung cancer cells.

### 3.4 DGCR5 and TUSC3 shared the same binding site for hsa‐miR‐873‐5p

**Figure 4:**
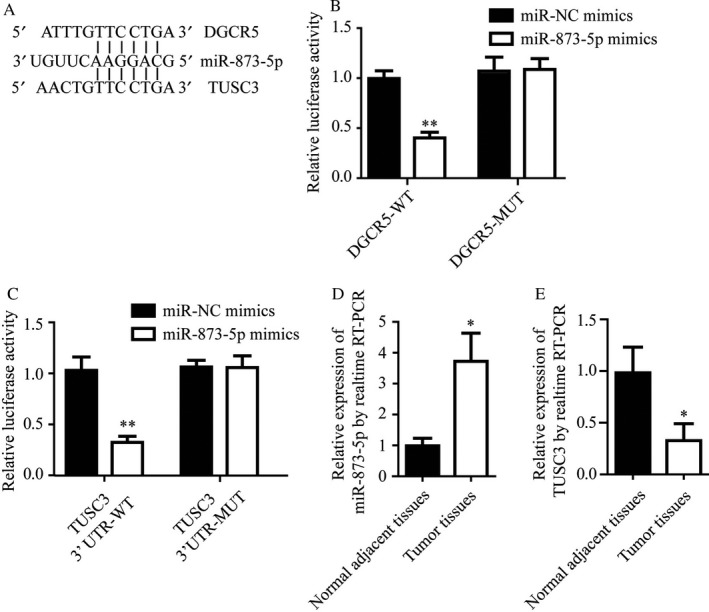
Both DGCR5 and TUSC3 binded to miR‐873‐5p. A, Analysis showed the potential binding sites of miR‐873‐5p to DGCR5 and TUSC3, with considerable sequence complementary in the indicated regions. B, miR‐873‐5p mimics reduced luciferase signal of reporter plasmid containing DGCR5 sequence but not DGCR5 mutant plasmid. *P < .01 compared with miR‐NC mimics. C, miR‐873‐5p mimics reduced luciferase signal of reporter plasmid containing TUSC3 3’UTR but not TUSC3 3’UTR‐mutant plasmid. *P < .01 compared with miR‐NC mimics. D, Elevation of miR‐873‐5p in lung tumor tissues compared with normal adjacent tissues was detected by RT‐qPCR. *P < .05 compared with normal adjacent tissues. E, Lower expression of TUSC3 in lung tumor tissues compared with normal adjacent tissues was detected by RT‐qPCR. *P < .05 compared with normal adjacent tissues

Through bioinformatics analysis on miRDB database, we predicted the miRNAs that might interact with DGCR5 (data not shown), hsa‐miR‐873‐5p got the highest score (85 score), and there were 2 specific binding sites between DGCR5 (801‐807 bp and 823‐829 bp) and hsa‐miR‐873‐5p. Thereafter, we predicted the mRNAs which could be bound by hsa‐miR‐873‐5p. Interestingly, there were conserved binding sites for miR‐873‐5p on both DGCR5 and 3’‐UTR of TUSC3 (49‐55 bp; Figure 4A).

To identify whether miR‐873‐5p directly bound to DGCR5, wild‐type (wt) or mutant (mut) DGCR5 cDNA sequence were cloned into pmirGLO vector, followed by cotransfection with miR‐873‐5p mimics or miR‐NC mimics and conduction of dual reporter luciferase in HEK293T cells. Results demonstrated that miR‐873‐5p mimics markedly inhibited the luciferase activity of pmirGLO‐DGCR5‐wt but not pmirGLO‐DGCR5‐mut (Figure 4B, *P* < .01).

To verify that TUSC3 was a direct target to miR‐873‐5p, TUSC3 3’‐UTR wild‐type or mutant 3’‐UTR was cloned into a luciferase reporter vector, followed by cotransfection of HEK293T cells with miR‐873‐5p mimics or miR‐NC mimics. Results indicated that miR‐873‐5p remarkably reduced the luciferase activity of TUSC3‐wt‐3’‐UTR but not TUSC3‐mut‐3’‐UTR (Figure 4C, *P* < .01).

For figuring out the roles of miR‐873‐5p and TUSC3 in lung cancer progression, the expression levels of miR‐873‐5p and TUSC3 in the neoplastic and non‐neoplastic tissues from patients with LC were detected by qRT‐PCR. We found that miR‐873‐5p expression status was significantly elevated in neoplastic tissues compared with non‐neoplastic tissues (Figure 4D, *P* < .01); while mRNA level of TUSC3 was notably downregulated in neoplastic tissues in comparison with non‐neoplastic tissues (Figure 4E, *P* < .01).

### 3.5 DGCR5 negatively regulated TUSC3 through hsa‐miR‐873‐5p

Next we sought to explore whether there was a regulatory relationship between DGCR5 and TUSC3.

**Figure 5:**
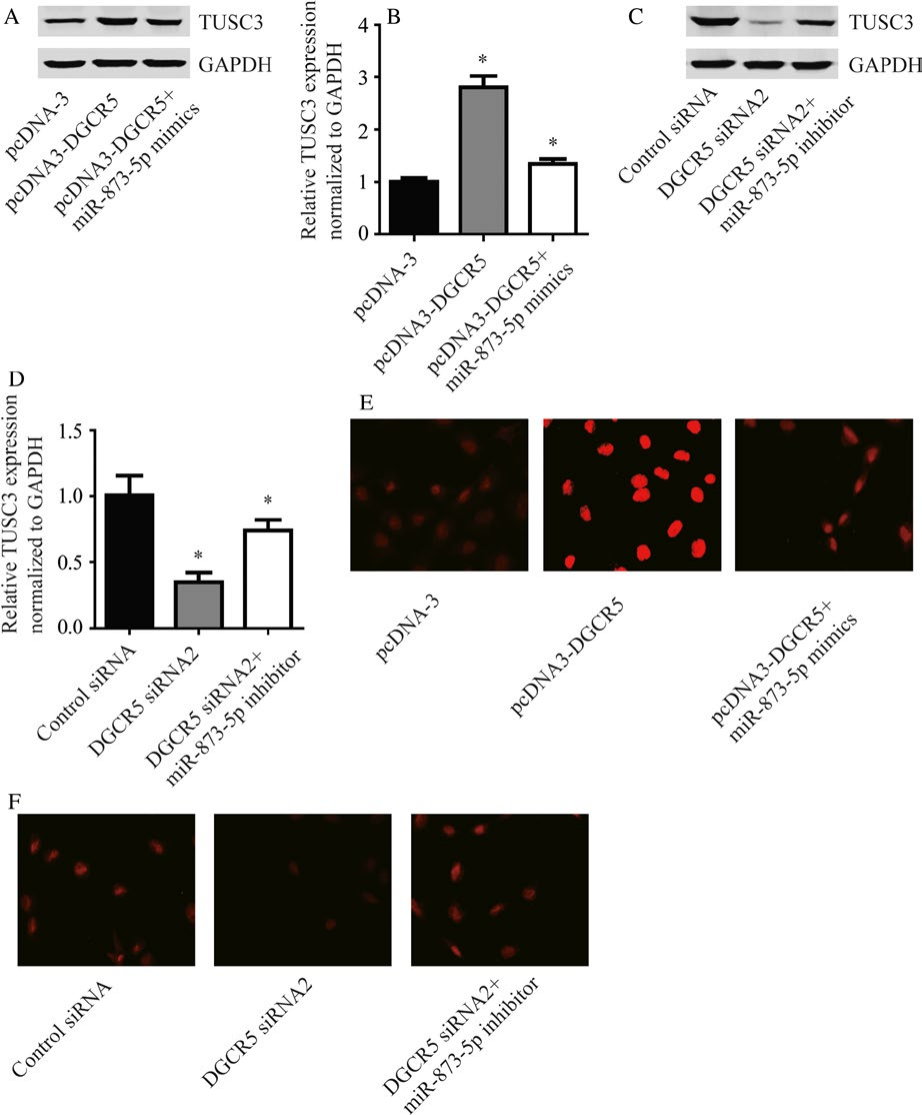
DGCR5 regulated TUSC3 via targeting miR‐873‐5p. A, The protein level of TUSC3 was increased in response to DGCR5 over‐expression and could be reversed by miR‐873‐5p mimics. B, Quantitative analysis of TUSC3 protein level in (A). C, The protein level of TUSC3 was decreased in response to DGCR5 siRNA2 and could be reversed by miR‐873‐5p inhibitor. D, Quantitative analysis of TUSC3 protein level in (C). E, Using immunofluorescence, significant increase of TUSC3 protein was observed in A549 cells with over‐expression of DGCR5 which could be reversed by miR‐873‐5p mimics. The red indicated TUSC3 protein. F, Using immunofluorescence, significant decrease of TUSC3 protein was observed in A549 treated with DGCR5 siRNA2 which could be reversed by miR‐873‐5p inhibitor. The red indicated TUSC3 protein

Results of Western blot exhibited that there was significant elevation of TUSC3 protein level in A549 cells transfected with pcDNA3‐DGCR5 than pcDNA3, while dramatic reduction was generated by cotransfection with pcDNA3.1‐DGCR5 and miR‐873‐5p mimics (Figure 5A,B, *P* < .01). Meanwhile, significant decline of TUSC3 protein level was caused by transfection of DGCR5 siRNA2 compared with DGCR5 control siRNA, and cotransfection with DGCR5 siRNA2 and miR‐873‐5p inhibitor rescued the decline predominantly (Figure 5C,D, *P* < .01).

Similarly, results of immunofluorescence were consistent with those of Western blot (Figure 5E‐H, *P* < .01). These data suggested that DGCR5 regulated TUSC3 through miR‐873‐5p.

### 3.6 DGCR5 inhibited tumor growth in nude mice

**Figure 6:**
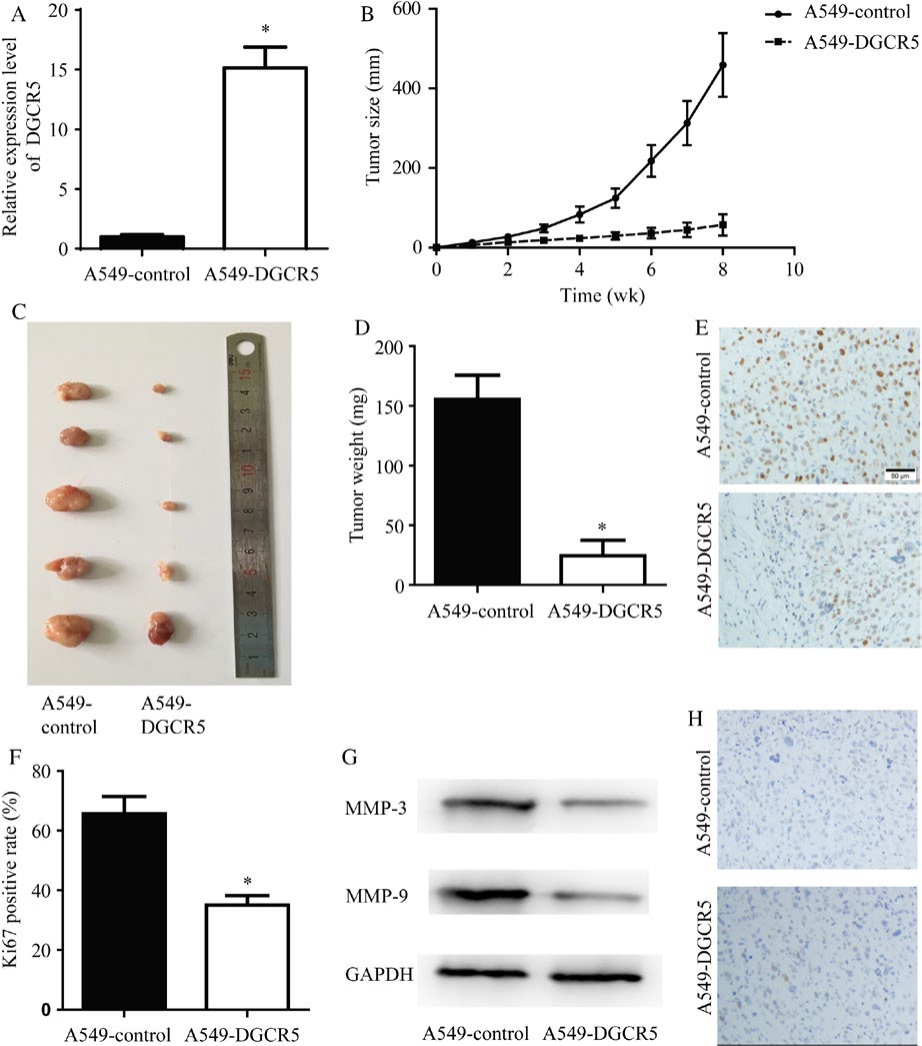
DGCR5 inhibited lung cancer growth in vivo. A, Elevated DGCR5 expression was detected in A549‐DGCR5 with stable over‐expression of DGCR5 compared with A549‐control group. **P < .01 compared with A549‐control. B‐D, The tumor growth of A549‐DGCR5 was greatly inhibited compared with A549‐control group. *P < .05 compared with A549‐control; **P < .01 compared with A549‐control. E and F, Representative images and quantitative analysis of Ki67 immunohistochemistry staining of tumors formed by A549‐control and A549‐DGCR5. G and H, The A549‐DGCR5 formed tumors showed a decrease of Ki67 positive staining rate. *P < .05 compared with A549‐control

To explore the role of DGCR5 in tumorigenesis in vivo, A549‐DGCR5 cells with stable overexpression of DGCR5 or A549‐control cells transfected with control vector were subcutaneous injected into nude mice (Figure 6A). Consistent with in vitro assays, elevation of DGCR5 greatly reduced tumor size, and the tumor weight of A549‐DGCR5 group was significantly lighter in comparison with A549‐control group (Figure 6B‐D).

Ki‐67 protein was a cellular marker for proliferation,^19^ consistently, IHC exhibited that protein level of Ki‐67 was significantly lower in A549‐DGCR5 group in comparison with A549‐control group (Figure 6E,F). Moreover, DGCR5 also lead to significant expression in migration and invasion‐related marker MMP‐3 and MMP‐9 (Figure 6G) in tumor samples. On the other hand, results of TUNEL staining between the 2 groups did not show significant differences (Figure 6H). These data further confirmed that low expression of DGCR5 contributed to cancer development in lung cancer.

## 4 DISCUSSION

The current therapies, including radiotherapy, chemotherapy, and the emerging target therapy, remain unsatisfactory for improving the therapeutic efficacy of patients with LC.^5^

Mounting evidences discovered the role of lncRNAs in various diseases, for instance, DGCR5 was first reported to be decreased in Huntington’s disease^20^; DGCR5 inhibited cell proliferation, migration via bounding to miR‐320a in pancreatic cancer.^18^ The finding in pancreatic cancer suggested DGCR5 as the tumor suppressor. We found that DGCR5 was downregulated in the tissues of patients with LC. In addition, we carried out in vitro experiments and also found that DGCR5 inhibited cell proliferation, migration, and invasion with no obvious effects on cell apoptosis of A549 cells, in vivo assay further confirm a tumor suppressor role for DGCR5 in LC evidenced by elevation of DGCR5 greatly reduced tumor size/weight and Ki67 protein expression in comparison with A549‐control group. During we carried out our work, Chen and his colleagues declared that DGCR5 suppressed proliferation and metastasis of LC cell lines H520 and H1299, which is consistent with our finding.^21^

Some lncRNAs function as sponges and regulate levels and activities of miRNAs.^12^, ^13^ miRNAs were primary regulators of gene expression via targeting 3’‐UTR of target genes^14^ and regulated biological processes in numerous cancers.^16^ In the current study, we indicated that DGCR5 shared miR‐873‐5p response element with TUSC3; elevated miR‐873‐5p expression status and downregulated TUSC3 mRNA level was discovered in neoplastic tissues in comparison with non‐neoplastic tissues.

Downregulated miR‐873‐5p was found in the hippocampus of memory‐impaired temporal lobe epilepsy rats^22^; hsa‐miR‐873‐5p was decreased in myometrium of primiparous women in comparison with that of multiparous women^23^; more interestingly, in NSCLC tissue, miR‐873‐5p was reported to be nearly 3 fold higher than that in control tissue,^24^ which implied the role of miR‐873‐5p in promoting lung cancer progression.

TUSC3 located on chromosomal band 8p22 and was primarily characterized as a tumor suppressor gene,^25^ which was also corroborated by Yu et al. in March 2017.^26^ Frequent inactivation or lost of TUSC3 occurred to numerous cancers including ovarian and pancreatic cancer.^27^, ^28^

Consequently, we performed Western blot and immunofluorescence to detect the influence of DGCR5 and miR‐873‐5p on TUSC3 expression. We found that there was significant elevation of TUSC3 protein level and immunofluorescence density in A549 cells transfected with pcDNA3.1‐DGCR5 than pcDNA3.1, while dramatic reduction was generated by cotransfection with pcDNA3.1‐DGCR5 and miR‐873‐5p mimics. The results in our study suggested TUSC3 as a tumor suppressor gene, which was in consistent with a recently published study.^29^

Therefore, the present study investigated the function of DGCR5 in LC and the interaction of DGCR5/miR‐873‐5p/TUSC3 in LC, eventually providing a novel therapeutic target for LC.

## CONFLICT OF INTEREST

None.
